# Quitting activity and use of cessation assistance reported by smokers in eight European countries: Findings from the EUREST-PLUS ITC Europe Surveys

**DOI:** 10.18332/tid/98912

**Published:** 2018-12-21

**Authors:** Karin Hummel, Gera E. Nagelhout, Geoffrey T. Fong, Constantine I. Vardavas, Sophia Papadakis, Aleksandra Herbeć, Ute Mons, Bas van den Putte, Ron Borland, Esteve Fernández, Hein de Vries, Ann McNeill, Shannon Gravely, Krzysztof Przewoźniak, Piroska Kovacs, Antigona C. Trofor, Marc C. Willemsen, on behalf of the EUREST-PLUS consortium

**Affiliations:** 1 Department of Health Promotion, Maastricht University, Maastricht, the Netherlands; 2 Department of Family Medicine, Maastricht University, Maastricht, the Netherlands; 3 IVO Research Institute, The Hague, the Netherlands; 4 Department of Psychology & School of Public Health and Health Systems, University of Waterloo, Waterloo, Canada; 5 Ontario Institute for Cancer Research, Toronto, Canada; 6 University of Crete (UoC), Heraklion, Greece; 7 European Network on Smoking and Tobacco Prevention (ENSP), Brussels, Belgium; 8 Division of Prevention and Rehabilitation, University of Ottawa Heart Institute, Ottawa, Canada; 9 Health Promotion Foundation, Warsaw, Poland; 10 UK Centre for Tobacco and Alcohol Studies, London, United Kingdom; 11 Department of Behavioural Science and Health, University College London, London, United Kingdom; 12 Cancer Prevention Unit & WHO Collaborating Centre for Tobacco Control, German Cancer Research Center (DKFZ), Heidelberg, Germany; 13 Department of Communication, University of Amsterdam (ASCoR), Amsterdam, the Netherlands; 14 Netherlands Expertise Center for Tobacco Control, Trimbos Institute, Utrecht, the Netherlands; 15 Cancer Council Victoria, Melbourne, Australia; 16 Tobacco Control Unit, Catalan Institute of Oncology (ICO), and Cancer Control and Prevention Group, Bellvitge Biomedical Research Institute (IDIBELL), L’Hospitalet, Catalonia, Spain; 17 School of Medicine and Health Sciences, University of Barcelona, Catalonia, Spain; 18 Addictions Department, Institute of Psychiatry, Psychology & Neuroscience, King’s College London, London, United Kingdom; 19 Maria Sklodowska-Curie Institute - Oncology Center, Warsaw, Poland; 20 Smoking or Health Hungarian Foundation (SHHF), Budapest, Hungary; 21 University of Medicine and Pharmacy ‘Grigore T. Popa’ Iasi, Iasi, Romania; 22 Aer Pur Romania, Bucharest, Romania

**Keywords:** survey, smoking cessation, socioeconomic differences

## Abstract

**INTRODUCTION:**

There is clear evidence that the use of cessation aids significantly increases the likelihood of successful smoking cessation. The aim of this study was to examine quitting activity and use of cessation aids among smokers from various European countries. Subgroup differences were also examined for sex, income, education, and age in each country.

**METHODS:**

Cross-sectional data were collected in 2016 from 10683 smokers in eight European countries participating in the ITC Project: England (n=3536), Germany (n=1003), Greece (n=1000), Hungary (n=1000), the Netherlands (n=1136), Poland (n=1006), Romania (n=1001), and Spain (n=1001). We measured quitting activity, including quit attempts in the previous 12 months and intention to quit, use of cessation aids (i.e. medication, quitlines, internet, local services, e-cigarettes), and whether respondents had received advice from health professionals about quitting and e-cigarettes.

**RESULTS:**

Quit attempts were most common in England (46.3%) and least common in Hungary (10.4%). Quit intention was highest in England and lowest in Greece. Use of e-cigarettes to quit was highest in England (51.6%) and lowest in Spain (5.0%). Use of cessation aids was generally low across all countries; in particular this was true for quitlines, internet-based support, and local services. Receiving health professional advice to quit was highest in Romania (56.5%), and lowest in Poland (20.8%); few smokers received advice about e-cigarettes from health professionals. No clear differences were found for sex and income groups. Across countries, smokers with lower education reported less quitting activity.

**CONCLUSIONS:**

Quitting activity and use of cessation methods were low in most countries. Greater quit attempts and use of cessation aids were found in England, where large investments in tobacco control and smoking cessation have been made. Health professionals are important for motivating smokers to quit and promoting the effectiveness of various methods, but overall, few smokers get advice to quit.

## INTRODUCTION

A total of 180 countries comprising 90% of the world’s population have united under their ratification of the WHO Framework Convention on Tobacco Control (FCTC) to implement strong evidence-based measures to reduce the harms of tobacco use and of secondhand smoke. As measures to significantly reduce demand for tobacco products are implemented, strategies to assist smokers in quitting are gaining greater importance. FCTC Article 14 calls upon Parties to implement effective measures to promote adequate treatment for tobacco dependence^1^. To date, however, compared to other Articles from the FCTC, implementation of Article 14 has progressed slowly; indeed, Article 14 has been referred to as the ‘neglected Article’^2-5^. Previous research has suggested that concerns about high costs for cessation treatment could be an important factor for the low implementation rate in several countries, particularly low-income countries^3,6^. However, a review has shown that there are several low-cost forms of treatment that can quickly be implemented^7^. One important first step to addressing the lack of progress on advancing support for cessation, as called for by Article 14, is to assess the use of smoking cessation assistance across countries and jurisdictions. This would enable an assessment of whether accessibility affects use of treatments and whether use can be enhanced.

The most effective method to stop smoking is a combination of cessation pharmacotherapy (such as varenicline, cytisine, nicotine replacement therapy or bupropion) and behavioral support delivered face-to-face^7-10^. A variety of other approaches to smoking cessation are available with modest evidence for effectiveness, such as electronic cigarettes (e-cigarettes), behavioral support provided remotely, (e.g. over the internet), and a range of other self-help interventions, such as books, leaflets or digital aids (e.g. SMS texting or smartphone-based interventions)^11-15^. In accordance with Article 14, evidence-based strategies for increasing quitting include the involvement of health care professionals in encouraging and assisting smokers in quitting, increasing availability and accessibility of cessation services, and integrating cessation services and cessation advice into existing health systems^16,17^. However, implementation of these measures varies significantly across countries^3^.

There are very few cross-country studies on the use of cessation aids. A study conducted by Borland et al.^18^ among smokers from 15 countries participating in the International Tobacco Control (ITC) Project showed that use of cessation support by smokers was generally quite low. However, this study used data collected from 2006–2009, and included only five European countries (UK, Ireland, Netherlands, Germany, France). More recent research using the Eurobarometer has noted that only slightly more than one in four smokers in Europe in 2017 had used any form of smoking cessation assistance^19^. The study by Borland et al.^18^ also demonstrates high variability in the use of cessation methods across countries, including European countries. For example, use of smoking cessation medication was reported by nearly 50% of smokers from the UK, but by less than 10% of smokers from Germany. This is likely as England fully reimburses the costs of cessation medication for some smokers whereas Germany does not provide reimbursement.

This article reports recent data on quitting activity, including quit attempts and intention to quit, and use of cessation assistance among smokers from eight European countries participating in the ITC Project: England, Germany, Greece, Hungary, the Netherlands, Poland, Romania, and Spain. In addition, we investigate whether there were differences in quitting activity and use of cessation aids between different sociodemographic groups (sex, income, education, age groups) in each country, as previous research has shown that smoking and cessation behaviors differ between these subgroups^20-23^.

Box 1 provides an overview of the different smoking cessation systems in 2016 in the European countries included in this study that are relevant for the current study. All countries had national guidelines for the treatment of tobacco dependence, and these guidelines were also widely used and disseminated, although dissemination was limited in Poland. Only England and the Netherlands had national mass media campaigns about smoking cessation implemented in 2016. Quitlines were available in all countries, except in Greece and only in a few regions in Spain. Cessation medication was reimbursed in the Netherlands, Romania, for some smokers in England, and in a few regions of Spain. All countries except Greece and Spain promoted cessation assistance, for example, by information that was printed on cigarette packs.

Box 1 **Overview of the cessation support systems in the eight European countries in 2016**

**Table:** 

*Country*	*National mass media campaign(s) about how to quit*	*Availability of quitline(s)*	*Reimbursement and subsidies of smoking cessation medications*	*Availability of national guidelines for the treatment of tobacco dependence*	*Use and dissemination of national guidelines, for example endorsed by key bodies*
England	Yes	Yes	Yes	Yes	Yes
Germany	No	Yes	No	Yes	Yes
Greece	No	No	No	Yes	Yes
Hungary	No	Yes	No	Yes	Yes
Netherlands	Yes	Yes	Yes	Yes	Yes
Poland	No	Yes	No	Yes***	Yes***
Romania	No	Yes	Yes	Yes	Yes
Spain	No	Yes†	Yes†	Yes	Yes
***Country***	***Promotion of cessation assistance, for example telephone number of quitline printed on cigarette packs***	Prevalence of current/ former smokers*	Prevalence of current/ former e-cigarette users*		Rank Tobacco Control Scale** (Range: 1-35, higher score = lower level of implementation of tobacco control policies)
England	Yes	17% / 18%	5% / 13%		1
Germany	Yes	25% / 18%	2% / 10%		33
Greece	No	37% / 17%	3% / 12%		31
Hungary	Yes	27% / 13%	1% / 8%		9
Netherlands	Yes	19% / 25%	2% / 13%		9
Poland	Yes	30% / 16%	1% / 12%		15
Romania	Yes	28% / 12%	0% / 9%		7
Spain	No	28% / 21%	1% / 11%		8

* Source: Eurobarometer28 March 2017.

** Source: Tobacco Control Scale29 2016.

*** Limited/not updated. †In a few regions.

## METHODS

### Design and sample

We used data collected in 2016 from eight European countries participating in the ITC Project^24-26^: England, Germany, Greece, Hungary, the Netherlands, Poland, Romania, and Spain. The data from six countries – Germany, Greece, Hungary, Poland, Romania, Spain – were collected in the ITC 6 European Country (6E) Survey as part of the EUREST-PLUS Project, funded by Horizon 2020, which aims to evaluate the impact of the European Tobacco Products Directive and WHO FCTC implementation in the EU^27^. The data from England and the Netherlands were collected as part of other ITC survey projects in Europe. Although the selection criteria, cohort design, and the survey questionnaire (described below) were all very closely linked across the eight countries, the sample composition and survey modality varied. As such, the ITC 6E Survey + ITC surveys in England and the Netherlands are collectively referred to in this paper as the ‘EURESTPLUS ITC Europe Surveys’.

The 6E Survey was conducted between 18 June and 12 September 2016 with interviews of 6011 adult cigarette smokers age 18 years or older selected from a sampling design conceptualized to create nationally representative cohorts in each of the six countries. The geographic strata were NUTS regions crossed with degree of urbanization (urban, intermediate, rural). Approximately 100 area clusters were sampled in each country, with the aim of obtaining 10 adult smokers per cluster. Clusters were allocated to strata proportionally to a population size with age 18 years and older. Within each cluster, household addresses were sampled using a random walk design. One randomly selected male smoker and one randomly selected female smoker were chosen for interview from a sampled household, where possible. Screening of households continued until the required number of smokers from the cluster had been interviewed. All interviews were conducted face-to-face by interviewers using tablets (CAPI), after written informed consent was obtained^30^. Individual response rates ranged from 70% in Germany to 93% in Hungary^30^. Ethics approval for data collection was provided for each national cohort by the corresponding local ethics committees.

Data from England were collected by the 2016 ITC Four-Country Smoking and Vaping Wave 1 (4CV1) Survey. This project is an expansion of the 2002– 2015 ITC Four-Country (ITC 4C) Project^31^. The 4CV1 sample was designed to be as representative as possible of smokers (e.g. age and sex) and consisted of re-contacted respondents from the ITC 4C cohort and new respondents from online commercial panels. The sample comprised the following cohorts: 1) recontact smokers and quitters living in England who participated in earlier waves of the ITC United Kingdom Survey, regardless of e-cigarette use; 2) newly recruited current smokers and recent quitters (quit smoking in the past 24 months), regardless of e-cigarette use; and 3) newly recruited current e-cigarette users (use at least weekly). In sampling, quotas obtained from national survey data for region crossed with male/female were applied^32^ to 2) and 3). Respondents were 4374 adults, which included 304 from the previous ITC cohort and 4070 new respondents. The response rate for new recruits was 15.2%. The survey was cleared for ethics by the Research Ethics Boards of the University of Waterloo and King’s College London.

Data from the Netherlands were collected as part of Wave 10 of the ITC Netherlands Survey, a cohort web survey that began in 2008. Data for Wave 10 were collected between 15 November and 31 December 2016. Respondents were 1696 adults age 15 years or older recruited as cigarette smokers, who were part of a probability-based web database^33^. The nationally representative sample included 1318 who had also responded in Wave 9 and 378 new respondents recruited to replenish dropouts^34,35^. The response rate was 67%. The survey was cleared for ethics by the Research Ethics Board of the University of Waterloo.

For the current study, we selected tobacco cigarette smokers age 18 years and older. Respondents were classified as smokers if they had smoked at least 100 cigarettes in their lifetime and were currently smoking cigarettes at least monthly. This resulted in the following sample sizes: n=3536 in England, n=1003 in Germany, n=1000 in Greece, n=1000 in Hungary, n=1136 in the Netherlands, n=1006 in Poland, n=1001 in Romania, and n=1001 in Spain.

### Measurements

#### Quitting activity

We asked respondents who had ever made a quit attempt about their quit attempts in the previous 12 months. The Netherlands and 6E Surveys asked: ‘Have you made an attempt to quit smoking in the last 12 months?’ (yes/no). In England, smokers were asked: ‘How many times, if any, have you tried to quit in the past 12 months?’; with response options: no attempt, one attempt, two attempts, three attempts or more. This was dichotomized into no attempt versus any attempt.

To measure intention to quit, we asked: ‘Are you planning to quit smoking…’; with the following response options: 1) within the next month, 2) within the next 6 months, 3) sometime in the future, beyond 6 months, and 4) or are you not planning to quit?^36^

#### Use of cessation assistance

Use of all cessation methods described below was only asked to smokers who had made a quit attempt in the previous 12 months.

First, we asked whether smokers had used any cessation pharmacotherapy during their previous quit attempt. The 6E Survey asked: ‘Which of the following products and services did you use as part of your last quit attempt? 1) Any type of nicotine replacement product, such as patches, gum, mouth spray etc., 2) varenicline or Chantix™ or Champix, 3) bupropion or Zyban or Wellbutrin, 4) Cytisine, Desmoxan, or Tabex’. This was dichotomized into use of any versus none of the products. In England, we asked: ‘Which of the following forms of help did you receive or use as part of your last quit attempt? 1) Any type of nicotine replacement product, such as patches, gum, mouth spray etc., 2) varenicline or Chantix or Champix, 3) bupropion or Zyban or Wellbutrin’. This was again recoded to indicate whether respondents had used any of the products versus none. In the Netherlands, we asked: ‘Have you used any stop-smoking medications, such as nicotine replacement therapies like nicotine gum or the patch, or other medications that require a prescription, such as Zyban in the last 12 months?’ (yes/no).

Second, we asked whether smokers had used a quitline. In the Netherlands, respondents were asked about the use of a telephone or quitline service in the previous 6 months, while smokers from the other countries were asked about the use of a telephone or quitline service as part of their previous quit attempt.

Third, we asked whether smokers had used the internet in the context of quitting, i.e. a website about quitting smoking. Smokers from the Netherlands were asked about use in the previous 6 months, while smokers from the other countries were asked about use of the internet as part of their previous quit attempt.

Fourth, we asked whether smokers had used local stop-smoking services such as clinics or specialists. Smokers from the Netherlands were asked about use in the previous 6 months, while smokers from the other countries were asked about use of the internet as part of their previous quit attempt.

Finally, we asked whether respondents had used an e-cigarette as part of their previous quit attempt.

#### Advice from health professionals

We asked respondents whether they had visited a doctor or health professional in the previous 6 months in the Netherlands, and the previous 12 months in the other countries. Respondents who answered ‘yes’ to this question were asked two follow-up questions.

We asked whether respondents received advice about quitting. In the Netherlands, this question was: ‘During any visit to the doctor or other health professional in the last 6 months, did you receive advice concerning possible ways to stop smoking?’. In the remaining countries, this question was: ‘During any visit with a doctor or health professional in the last 12 months, did you receive advice to quit smoking?’. Furthermore, we asked whether respondents had spoken about e-cigarettes with health professionals: ‘On any visit to the doctor or health professional in the last 12 months, did the doctor or health professional talk to you about e-cigarettes?’.

### Statistical analyses

Data were analyzed with SPSS 23.0. We analyzed data for each country separately and calculated percentages with 95% confidence intervals for proportions. We performed subgroup analyses according to sex, income, education, and age, by calculating percentages with 95% confidence intervals. All statistical estimates presented were weighted for sex and age to make the data more representative for the population of smokers in each country. More information about the methods, such as sampling design and weight constructions, can be found elsewhere^27,30,32,35^. The 6E data were analyzed using the Complex Samples package to take the complex sampling design into account. Missing data were excluded on a case-by-case basis.

## RESULTS

### Quitting behavior, use of cessation assistance and advice from health professional

Table 1 shows the percentages of smokers in each of the eight countries who reported their intention to quit, having made a quit attempt in the past 12 months and if so, whether they had used specific cessation methods (pharmacotherapy, quitlines, internet, smoking cessation services, e-cigarettes), and whether they had received advice about quitting from and talked about e-cigarettes with health professionals.

**Table 1:** Reported quitting activity and use of cessation assistance in 2016 across eight European countries (weighted results)

	*England (n=3536) % (95%CI)*	*Germany (n=1003) % (95%CI)*	*Greece (n=1000) % (95%CI)*	*Hungary (n=1000) % (95%CI)*	*Netherlands (n=1136) % (95%CI)*	*Poland (n=1006) % (95%CI)*	*Romania (n=1001) % (95%CI)*	*Spain (n=1001) % (95%CI)*
Made quit attempt in past 12 months	46.3 (45.3-47.3)	17.1 (14.1-20.5)	15.1 (12.2-18.4)	10.4 (8.3-13.0)	31.5 (28.3-35.0)	16.2 (13.0-20.1)	27.1 (23.6-30.8)	17.7 (14.2-21.7)
Intends to quit								
Within 1 month	14.3 (12.7-16.0)	3.9 (2.7-5.8)	2.0 (1.2-3.2)	2.2 (1.3-3.6)	7.5 (5.5-10.0)	4.3 (2.7-6.6)	6.9 (5.2-9.1)	4.8 (3.2-7.2)
Within 6 months	25.9 (24.0-28.0)	7.5 (5.5-10.1)	6.2 (4.7-8.2)	8.2 (6.3-10.7)	22.7 (19.5-26.2)	9.7 (7.2-12.9)	9.3 (7.1-12.2)	8.2 (6.4-10.4)
Beyond 6 months	34.0 (31.9-36.2)	46.2 (41.5-51.0)	32.3 (28.2-36.6)	21.5 (18.3-25.1)	50.9 (47.0-54.9)	27.3 (23.5-31.4)	37.4 (32.3-42.7)	23.5 (19.1-28.6)
Not at all	25.7 (23.8-27.7)	42.4 (36.8-48.1)	59.5 (54.8-64.1)	68.1 (63.7-72.1)	18.9 (16.0-22.3)	58.7 (53.5-63.7)	46.4 (40.5-52.4)	63.5 (58.3-68.4)
Used medication*	16.1 (15.4-16.8)	1.9 (1.2-3.0)	1.5 (0.8-2.7)	1.7 (0.9-3.2)	7.5 (7.0-8.0)	4.7 (3.3-6.6)	3.1 (1.9-5.0)	2.3 (1.4-3.9)
Used quitline**	3.0 (2.7-3.3)	1.3 (0.3-5.6)	Not applicable	0	1.7 (1.4-2.0)	1.8 (0.5-6.2)	1.1 (0.2-7.1)	0
Used internet**	10.9 (9.0-13.1)	4.8 (1.8-12.2)	0.4 (0.1-2.6)	2.7 (0.4-16.9)	5.7 (5.2-6.2)	5.7 (2.6-12.1)	3.2 (1.0-9.8)	0
Used smoking cessation service**	8.4 (6.8-10.3)	1.0 (0.2-4.0)	1.2 (0.3-4.7)	0.6 (0.1-4.2)	4.0 (3.6-4.4)	2.1 (0.8-5.3)	0.2 (0.0-1.4)	2.2 (0.5-8.8)
Used e-cigarettes to quit	51.6 (47.9-55.2)	15.9 (10.7-23.0)	28.7 (20.7-38.3)	16.2 (9.6-26.1)	43.8 (34.9-53.1)	13.0 (7.7-21.1)	11.0 (6.5-18.2)	5.0 (2.3-10.5)
Received advice about quitting from health professional during visit***	38.3 (35.7-40.9)	39.3 (32.8-46.2)	53.0 (41.9-63.8)	21.7 (16.2-28.3)	21.8 (21.0-22.6)	20.8 (16.8-25.5)	56.5 (50.7-62.1)	45.7 (39.1-52.5)
Talked about e-cigarettes with health professional	6.7 (5.6-7.9)	0.6 (0.2-1.9)	4.7 (2.7-8.1)	9.8 (5.2-17.6)	6.3 (3.9-9.8)	2.7 (1.0-7.2)	8.3 (5.5-12.2)	2.6 (1.3-5.1)

* Netherlands: in the last 12 months. Other countries: as part of your last quit attempt.

** Netherlands: in the last 6 months. Other countries: as part of your last quit attempt.

*** Netherlands: in the last 6 months. Other countries: in the last 12 months.

There was substantial variation across countries in the percentage of smokers who reported making quit attempts: the highest was in England, where 46.3% of smokers reported making a quit attempt in the past 12 months; the lowest was in Hungary, where 10.4% of smokers reported attempting to quit in the past 12 months. The highest intention to quit within the next month was also found in England with 14.3%, while the lowest intention was reported by smokers from Greece, where 2.0% intended to quit within the next month.

Also, use of smoking cessation medication varied widely across countries. England was the country where smokers were most likely to report using smoking cessation medication as part of their previous quit attempt (16.1% of smokers); Greece was the country where smokers were least likely to report using smoking cessation medication (1.5% of smokers who had attempted to quit). Use of quitlines, the internet and smoking cessation services were in general low across all countries. The highest use of a cessation quitline was found in England (3.0%), while not a single smoker from our samples in Hungary and Spain used a quitline (no quitlines are available in Greece). Use of the internet in the context of quitting was again highest in England (10.9%), while no smokers from our sample in Spain reported using the internet for assistance in quitting. Use of smoking cessation services was also highest in England (8.4%) and lowest in Romania (0.2%). E-cigarettes were the most popular quit smoking aid used across all countries although the prevalence varied substantially across countries: it was highest in England (51.6%) and lowest in Spain (5.0%).

Smokers in Romania were most likely to receive advice from health professionals about quitting during a visit in the previous 12 months (56.5%); smokers in Poland were least likely to receive advice (20.8%). Few smokers reported being spoken to about e-cigarettes by health professionals across countries. This was reported most often by smokers from Hungary (9.8%), and least often by smokers from Germany (0.6%). A country ranking of the examined domains can be found in Supplementary Table S1.

### Subgroup analyses

We repeated the analyses by country for subgroups according to sex (Table 2), income (low, moderate, high; Table 3), education (low, moderate, high; Table 4) and age (Table 5). There were no consistent patterns when the results for men were compared with the results for women. In England and Germany, more men than women had no intention to quit at all. Furthermore, in England, more women than men used smoking cessation medication. In addition, in the Netherlands, more men than women reported having received advice about quitting from health professionals.

**Table 2:** Stratified analyses by sex on reported quitting activity and use of cessation assistance in 2016 across eight European countries (weighted results)

	*England (n=3536) % (95%CI)*	*Germany (n=1003) % (95%CI)*	*Greece (n=1000) % (95%CI)*	*Hungary (n=1000) % (95%CI)*	*Netherlands (n=1136) % (95%CI)*	*Poland (n=1006) % (95%CI)*	*Romania (n=1001) % (95%CI)*	*Spain (n=1001) % (95%CI)*
**Made quit attempt in past 12 months**								
Male	43.4 (42.4-44.4)	14.8 (11.4-19.1)	15.1 (11.6-19.4)	9.1 (6.5-12.6)	32.8 (28.2-37.9)	15.9 (12.2-20.6)	26.2 (22.1-30.7)	18.7 (14.5-23.8)
Female	49.8 (48.8-50.8)	20.6 (16.5-25.4)	15.0 (11.0-20.2)	12.3 (9.2-16.2)	29.7 (25.7-34.1)	16.6 (12.9-21.2)	28.4 (23.5-33.8)	16.3 (12.4-21.1)
**Intends to quit**								
**Within 1 month**								
Male	13.6 (11.5-16.0)	3.0 (1.7-5.3)	2.2 (1.3-3.8)	2.3 (1.1-4.6)	7.7 (5.0-11.7)	4.7 (2.6-8.7)	7.5 (5.4-10.5)	4.1 (2.6-6.4)
Female	15.1 (12.9-17.7)	5.4 (3.5-8.2)	1.7 (0.8-3.6)	2.0 (1.1-3.7)	7.1 (4.8-10.3)	3.7 (2.4-5.6)	6.0 (3.9-9.3)	5.8 (3.4-9.9)
**Within 6 months**								
Male	26.2 (23.4-29.1)	6.9 (4.7-10.1)	6.3 (4.4-8.8)	7.3 (5.1-10.3)	24.0 (19.3-29.3)	9.2 (6.6-12.8)	8.0 (5.6-11.4)	8.5 (6.1-11.6)
Female	25.7 (22.9-28.6)	8.4 (5.9-11.8)	6.2 (4.1-9.2)	9.6 (6.7-13.6)	20.9 (17.0-25.3)	10.3 (7.3-14.3)	11.2 (7.5-16.3)	7.7 (4.8-12.3)
**Beyond 6 months**								
Male	31.2 (28.4-34.3)	42.7 (36.2-49.6)	31.2 (26.4-36.5)	19.8 (15.5-25.0)	48.5 (43.0-54.1)	26.5 (21.6-32.0)	35.8 (30.1-42.0)	22.3 (17.2-28.4)
Female	37.3 (34.2-40.5)	51.7 (46.6-56.7)	33.5 (28.2-39.2)	24.1 (18.6-30.6)	54.4 (49.1-59.5)	28.4 (23.4-34.0)	39.6 (33.3-46.3)	25.1 (20.0-31.0)
**Not at all**								
Male	29.0 (26.3-31.9)	47.4 (40.6-54.2)	60.3 (54.5-65.8)	70.6 (64.9-75.7)	19.8 (15.7-24.7)	59.6 (53.4-65.5)	48.6 (41.4-55.9)	65.1 (59.1-70.7)
Female	21.9 (19.4-24.6)	34.5 (29.1-40.4)	58.6 (52.4-64.6)	64.3 (58.2-70.0)	17.7 (13.9-22.3)	57.6 (51.8-63.3)	43.2 (36.9-49.8)	61.3 (55.0-67.3)
Used medication*								
Male	14.4 (13.7-15.1)	1.0 (0.5-2.1)	0.7 (0.3-2.2)	1.4 (0.5-4.0)	7.4 (6.9-7.9)	3.8 (2.3-6.2)	3.2 (1.7-5.8)	2.8 (1.4-5.4)
Female	18.1 (17.3-18.9)	3.2 (1.9-5.3)	2.3 (1.1-4.7)	2.2 (0.9-5.0)	7.7 (7.2-8.2)	5.8 (3.7-9.0)	2.9 (1.4-6.1)	1.7 (0.9-3.5)
Used quitline**								
Male	4.3 (3.9-4.7)	2.5 (0.6-10.3)	Not applicable	0	1.9 (1.6-2.2)	1.0 (0.1-7.0)	0	0
Female	1.6 (1.4-1.8)	0	Not applicable	0	1.4 (1.2-1.6)	2.7 (0.5-12.3)	2.5 (0.3-16.0)	0
Used internet**								
Male	14.4 (11.4-18.1)	6.6 (2.2-18.2)	0	5.3 (0.8-29.2)	5.3 (4.9-5.7)	7.7 (2.9-18.9)	3.3 (0.7-15.2)	0
Female	7.3 (5.5-9.8)	2.9 (0.8-10.0)	0.8 (0.1-5.5)	0	6.3 (5.8-6.8)	3.3 (1.1-9.9)	3.0 (0.6-14.7)	0
Used smoking cessation service**								
Male	10.0 (7.6-13.1)	0.8 (0.1-5.3)	1.1 (0.1-7.3)	0	3.3 (2.9-3.7)	0	0	3.6 (0.9-13.8)
Female	6.7 (4.9-9.2)	1.2 (0.2-8.1)	1.3 (0.2-8.6)	1.2 (0.2-8.3)	5.0 (4.6-5.4)	4.5 (1.8-10.8)	0.5 (0.1-3.1)	0
**Used e-cigarettes to quit**								
Male	51.2 (45.9-56.6)	17.1 (9.8-28.2)	32.9 (21.9-46.1)	18.7 (9.3-34.1)	42.1 (29.5-55.8)	16.6 (9.1-28.4)	11.4 (5.5-22.3)	5.7 (1.9-15.4)
Female	51.9 (46.9-56.9)	14.5 (9.5-21.6)	24.0 (13.1-39.7)	13.6 (5.4-30.3)	46.2 (35.0-57.8)	8.8 (4.2-17.5)	10.6 (5.3-20.1)	4.0 (1.4-10.3)
Received advice about quitting from health professional during visit***								
Male	42.0 (38.3-45.9)	41.2 (32.8-50.1)	57.3 (49.4-64.9)	24.7 (17.8-33.3)	23.9 (23.1-24.7)	23.6 (18.1-30.2)	55.1 (47.5-62.5)	52.1 (43.1-61.0)
Female	34.8 (31.4-38.3)	37.0 (29.4-45.4)	49.3 (37.6-61.2)	18.0 (12.8-24.6)	19.4 (18.6-20.2)	18.6 (13.7-24.7)	58.0 (49.7-65.8)	38.4 (31.7-45.7)
**Talked about e-cigarettes with health professional**								
Male	9.1 (7.3-11.3)	0	4.7 (1.9-11.1)	10.7 (5.6-19.5)	6.2 (3.0-12.4)	3.7 (0.9-14.5)	10.7 (6.4-17.3)	3.5 (1.7-7.1)
Female	4.4 (3.2-5.9)	1.4 (0.4-4.3)	4.7 (2.4-9.1)	8.6 (4.1-17.3)	6.3 (3.6-10.7)	1.9 (0.8-4.3)	5.6 (1.6-17.6)	1.8 (0.7-4.4)

* Netherlands: in the last 12 months. Other countries: as part of your last quit attempt.

** Netherlands: in the last 6 months. Other countries: as part of your last quit attempt.

*** Netherlands: in the last 6 months. Other countries: in the last 12 months.

**Table 3:** Stratified analyses by income on reported quitting activity and use of cessation assistance in 2016 across eight European countries (weighted results)

	*England (n=3536) % (95%CI)*	*Germany (n=1003) % (95%CI)*	*Greece (n=1000) % (95%CI)*	*Hungary (n=1000) % (95%CI)*	*Netherlands (n=1136) % (95%CI)*	*Poland (n=1006) % (95%CI)*	*Romania (n=1001) % (95%CI)*	*Spain (n=1001) % (95%CI)*
**Made quit attempt in past 12 months**								
Low	42.8 (41.8-43.8)	16.7 (13.0-21.3)	12.7 (8.4-18.6)	8.7 (5.4-13.8)	39.5 (31.4-48.3)	12.6 (8.3-18.6)	25.5 (19.1-33.0)	20.7 (14.7-28.5)
Moderate	46.0 (45.0-47.0)	18.6 (14.3-23.7)	15.1 (11.8-19.0)	9.0 (6.0-13.4)	31.4 (25.1-38.4)	16.9 (12.6-22.4)	30.1 (25.1-35.5)	16.8 (12.7-21.8)
High	49.1 (48.1-50.1)	18.1 (13.3-24.2)	19.1 (12.8-27.5)	8.7 (4.9-15.1)	35.3 (29.3-41.7)	16.1 (11.3-22.3)	24.2 (17.2-32.9)	13.5 (7.2-23.9)
**Intends to quit**								
**Within 1 month**								
Low	13.2 (10.3-16.7)	3.2 (1.6-6.3)	1.3 (0.4-4.2)	1.3 (0.6-2.9)	9.6 (4.8-18.5)	8.2 (4.2-15.5)	6.9 (3.9-11.9)	4.5 (2.5-7.9)
Moderate	11.2 (8.9-14.0)	5.5 (3.4-8.5)	2.1 (1.1-3.7)	1.8 (0.8-3.9)	5.3 (2.9-9.5)	4.0 (2.1-7.2)	5.8 (3.9-8.4)	7.7 (4.1-14.2)
High	16.7 (14.0-19.7)	4.2 (2.4-7.2)	1.3 (0.2-7.6)	3.7 (1.6-8.4)	10.8 (7.1-16.0)	4.9 (2.0-11.6)	7.2 (4.2-12.1)	4.8 (1.4-15.0)
**Within 6 months**								
Low	24.1 (20.2-28.6)	7.5 (4.1-13.4)	6.4 (3.8-10.6)	9.7 (6.1-15.0)	17.6 (11.9-25.3)	11.4 (7.4-17.1)	8.1 (5.2-12.6)	10.9 (5.7-20.0)
Moderate	26.6 (23.1-30.4)	7.4 (4.4-12.2)	6.1 (4.1-9.1)	8.3 (5.1-13.1)	21.9 (15.8-29.4)	9.9 (6.3-15.2)	9.1 (6.2-13.3)	6.4 (3.7-10.7)
High	28.3 (25.1-31.8)	9.2 (6.0 -13.8)	6.1 (2.4-14.3)	6.7 (4.2-10.6)	30.1 (23.9-37.1)	11.0 (7.0-16.9)	11.0 (7.3-16.2)	13.1 (6.5-24.5)
**Beyond 6 months**								
Low	31.4 (27.3-35.9)	40.9 (34.0-48.1)	31.8 (25.3-39.0)	17.2 (11.3-25.1)	49.8 (40.4-59.1)	23.8 (17.2-31.8)	35.7 (27.0-45.4)	20.9 (14.2-29.5)
Moderate	35.8 (31.9-39.8)	46.9 (40.3-53.7)	33.6 (28.2-39.5)	20.2 (15.8-25.5)	57.2 (49.3-64.7)	32.7 (26.2-39.8)	40.5 (34.4-46.9)	21.6 (16.0-28.5)
High	34.7 (31.2-38.3)	51.0 (43.0-58.8)	46.0 (34.7-57.8)	27.7 (21.2-35.4)	46.3 (39.6-53.2)	34.0 (25.7-43.3)	35.3 (27.0-44.7)	17.0 (9.8-28.1)
**Not at all**								
Low	31.3 (27.0-35.9)	48.4 (40.9-56.1)	60.5 (53.0-67.6)	71.9 (63.2-79.2)	23.0 (16.0-31.7)	56.6 (47.4-65.4)	49.2 (39.2-59.3)	63.8 (54.0-72.5)
Moderate	26.5 (23.2-30.1)	40.2 (33.1-47.8)	58.2 (52.2-64.0)	69.7 (62.4-76.2)	15.7 (10.9-22.0)	53.5 (46.2-60.6)	46.6 (37.8-51.6)	64.2 (56.6-71.2)
High	20.3 (17.6-23.3)	35.6 (27.6-44.5)	46.6 (35.9-57.7)	61.8 (53.6-69.4)	12.8 (9.2-17.5)	50.1 (41.0-59.2)	46.5 (36.5-56.7)	65.1 (51.8-76.3)
Used medication*								
Low	17.7 (17.0-18.4)	1.5 (0.5-4.0)	0.4 (0.1-2.9)	1.8 (0.6-5.6)	7.2 (6.7-7.7)	4.6 (2.5-8.4)	1.3 (0.6-2.6)	2.1 (1.0-4.2)
Moderate	16.3 (15.6-17.0)	2.3 (1.1-4.7)	1.3 (0.5-3.3)	2.2 (0.8-5.9)	6.4 (5.9-6.9)	6.3 (4.1-9.7)	4.1 (2.5-6.9)	3.4 (1.9-6.2)
High	16.3 (15.6-17.0)	1.7 (0.8-3.5)	0	2.7 (0.7-9.6)	8.9 (8.3-9.5)	4.0 (1.9-8.0)	1.8 (0.5-6.5)	3.6 (0.8-14.0)
Used quitline**								
Low	1.3 (1.1-1.5)	3.2 (0.5-19.0)	Not applicable	0	2.4 (2.1-2.7)	4.7 (0.7-27.1)	0	0
Moderate	2.1 (1.8-2.4)	1.1 (0.2-6.8)	Not applicable	0	2.5 (2.2-2.8)	3.5 (0.7-15.7)	0	0
High	4.1 (3.7-4.5)	0	Not applicable	0	1.1 (0.9-1.3)	0	4.0 (0.5-24.6)	0
Used internet**								
Low	11.0 (7.3-16.4)	5.6 (0.8-29.4)	0	0	6.1 (5.6-6.6)	14.9 (5.5-34.6)	0	0
Moderate	7.2 (5.1-10.0)	7.1 (1.8-24.3)	0	0	5.8 (5.3-6.3)	7.7 (2.5-21.1)	0.5 (0.1-3.2)	0
High	13.0 (9.9-17.0)	2.2 (0.6-7.8)	3.2 (0.4-21.3)	13.9 (2.1-55.5)	5.6 (5.1-6.1)	0	5.6 (1.2-22.9)	0
Used smoking cessation service**								
Low	12.0 (8.3-17.2)	3.4 (0.8-13.7)	0	0	4.8 (4.4-5.2)	0	0	0
Moderate	8.2 (5.7-11.6)	0	2.2 (0.5-8.5)	0	2.9 (2.6-3.2)	3.7 (1.1-12.3)	0.4 (0.1-2.8)	1.3 (0.2-8.8)
High	6.7 (4.6-9.7)	0	0	3.0 (0.4-19.6)	5.8 (5.3-6.3)	1.6 (1.1-2.2)	0	0
**Used e-cigarettes to quit**								
Low	47.3 (39.9-54.9)	19.6 (11.1-32.3)	10.4 (5.8-18.0)	0	32.0 (18.0-50.2)	6.0 (0.9-32.1)	6.7 (2.2-18.5)	2.2 (0.3-13.7)
Moderate	56.4 (49.7-62.9)	5.6 (2.1-14.4)	27.2 (17.6-39.6)	14.7 (6.6-29.6)	47.6 (29.6-66.3)	12.7 (5.7-25.9)	10.0 (5.9-16.5)	3.4 (0.8-13.5)
High	52.0 (46.0-57.8)	22.1 (12.6-35.9)	42.1 (21.7-65.7)	13.9 (5.1-32.7)	46.9 (31.1-63.4)	18.0 (6.8-39.7)	14.9 (4.6-39.0)	0
Received advice about quitting from health professional during visit***								
Low	40.4 (35.3-45.7)	40.8 (31.1-51.4)	44.7 (29.6-60.8)	25.3 (17.3-35.4)	22.1 (21.3-22.9)	21.4 (14.7-30.1)	66.6 (53.7-77.4)	52.9 (41.6-64.0)
Moderate	37.7 (33.2-42.5)	33.1 (25.4-41.9)	54.0 (46.0-61.8)	23.5 (13.4-37.8)	27.0 (26.1-27.9)	25.0 (18.9-32.4)	53.6 (44.5-62.5)	41.4 (31.2-52.4)
High	39.7 (35.4-44.0)	41.6 (31.2-52.8)	41.9 (27.9-57.4)	21.8 (11.9-36.5)	20.1 (19.3-20.9)	9.1 (3.4-22.5)	54.1 (42.2-65.6)	35.7 (18.6-57.3)
**Talked about e-cigarettes with health professional**								
Low	5.1 (3.4-7.6)	0.5 (0.4-0.6)	0	8.0 (4.0-15.7)	4.6 (1.7-11.9)	4.4 (1.8-10.2)	6.2 (2.2-16.4)	3.1 (0.9-9.4)
Moderate	7.7 (5.5-10.7)	0	5.8 (3.0-11.0)	14.6 (5.4-33.8)	11.8 (5.9-22.2)	2.5 (0.5-12.0)	7.0 (3.4-13.8)	2.8 (1.0-7.8)
High	7.4 (5.8-9.4)	0.7 (0.5-0.8)	4.9 (0.6-29.1)	8.4 (3.5-18.9)	3.9 (1.6-9.6)	5.4 (0.8-29.1)	10.9 (4.2-25.5)	0

* Netherlands: in the last 12 months. Other countries: as part of your last quit attempt.

** Netherlands: in the last 6 months. Other countries: as part of your last quit attempt.

*** Netherlands: in the last 6 months. Other countries: in the last 12 months.

**Table 4:** Stratified analyses by education on reported quitting activity and use of cessation assistance in 2016 across eight European countries (weighted results)

	*England (n=3536) % (95%CI)*	*Germany (n=1003) % (95%CI)*	*Greece (n=1000) % (95%CI)*	*Hungary (n=1000) % (95%CI)*	*Netherlands (n=1136) % (95%CI)*	*Poland (n=1006) % (95%CI)*	*Romania (n=1001) % (95%CI)*	*Spain (n=1001) % (95%CI)*
**Made quit attempt in past 12 months**								
Low	46.4 (45.4-47.4)	17.2 (13.4-21.9)	12.2 (8.7-16.8)	9.3 (6.8-12.5)	24.0 (18.4-30.8)	16.0 (9.4-25.8)	29.1 (22.1-37.2)	14.5 (10.3-19.9)
Moderate	45.8 (44.8-46.8)	16.3 (12.6-20.9)	13.8 (10.5-18.1)	10.4 (7.0-15.2)	32.3 (27.6-37.5)	14.7 (11.4-18.7)	27.3 (23.0-32.0)	19.6 (14.8-25.4)
High	48.7 (47.7-49.7)	19.1 (14.4-24.7)	21.6 (14.8-30.4)	22.6 (13.5-35.3)	35.7 (29.6-42.2)	27.5 (19.8-36.9)	22.2 (13.6-33.9)	24.4 (14.6-37.7)
**Intends to quit**								
**Within 1 month**								
Low	12.3 (10.1-14.9)	4.3 (2.6-7.0)	1.8 (0.8-4.0)	1.8 (0.9-3.6)	4.7 (2.2-9.6)	3.7 (1.5-9.2)	4.0 (2.1-7.8)	3.5 (2.0-6.0)
Moderate	14.1 (11.9-16.5)	3.0 (1.8-5.2)	0.7 (0.2-1.8)	2.0 (0.8-4.5)	6.2 (3.8-10.0)	4.2 (2.5-6.8)	8.8 (6.4-11.9)	5.8 (3.6-9.3)
High	17.4 (14.6-20.4)	6.6 (2.3-17.6)	5.4 (2.9-9.7)	7.4 (3.3-16.1)	10.8 (6.9-16.4)	6.5 (2.4-16.2)	2.6 (0.8-8.3)	6.6 (2.8-14.6)
**Within 6 months**								
Low	22.6 (19.7-25.7)	5.8 (3.7-9.0)	4.9 (2.8-8.4)	7.7 (5.4-10.8)	16.7 (10.7-25.0)	5.3 (2.4-11.2)	10.7 (6.5-17.1)	6.7 (4.2-10.7)
Moderate	27.0 (24.2-30.0)	9.6 (6.4-14.2)	6.7 (4.7-9.5)	8.1 (5.0-12.8)	20.2 (15.9-25.2)	9.9 (7.1-13.7)	8.1 (5.8-11.1)	8.5 (6.5-11.0)
High	27.1 (23.9-30.6)	6.6 (2.5-16.2)	6.4 (3.7-10.8)	14.2 (8.5-22.8)	29.8 (23.7-36.9)	13.4 (7.9-21.6)	12.6 (8.0-19.3)	14.1 (6.7-27.3)
**Beyond 6 months**								
Low	33.7 (30.4-37.2)	42.8 (36.5-49.4)	29.2 (23.1-36.2)	20.9 (16.6-25.9)	48.7 (40.8-56.7)	23.9 (16.1-33.9)	31.5 (23.7-40.5)	20.1 (14.6-26.9)
Moderate	35.2 (32.2-38.3)	48.1 (41.7-54.5)	29.7 (24.6-35.3)	23.8 (18.4-30.1)	55.3 (49.5-61.0)	28.0 (23.7-32.7)	39.2 (33.3-45.4)	25.8 (19.7-33.1)
High	31.7 (28.2-35.4)	55.6 (46.0-64.9)	43.1 (35.2-51.5)	17.1 (11.6-24.5)	46.2 (39.3-53.3)	29.1 (20.5-39.4)	41.9 (31.5-53.0)	28.5 (18.9-40.4)
**Not at all**								
Low	31.4 (28.2-34.8)	47.0 (40.2-53.9)	64.1 (57.6-70.1)	69.7 (64.2-74.7)	29.9 (23.3-37.6)	67.1 (55.8-76.6)	53.8 (44.7-62.7)	69.7 (62.3-76.3)
Moderate	23.8 (21.2-26.6)	39.3 (32.2-46.8)	63.0 (57.1-68.5)	66.2 (59.8-72.0)	18.3 (14.0-23.5)	58.0 (51.7-64.0)	43.9 (37.3-50.8)	59.9 (52.9-66.5)
High	23.8 (20.8-27.2)	31.2 (24.6-38.6)	45.1 (36.1-54.4)	61.3 (48.2-72.9)	13.1 (8.9-18.9)	51.1 (40.8-61.3)	42.9 (33.1-53.4)	50.8 (37.8-63.6)
Used medication*								
Low	16.3 (15.6-17.0)	1.5 (0.7-3.2)	0.9 (0.3-2.6)	1.0 (0.3-3.6)	7.6 (7.1-8.1)	3.6 (1.4-8.7)	3.5 (1.7-7.0)	1.2 (0.5-2.9)
Moderate	15.8 (15.1-16.5)	2.2 (1.2-4.2)	1.2 (0.5-3.3)	2.2 (1.0-4.7)	7.7 (7.2-8.2)	5.1 (3.6-7.1)	3.1 (1.6-5.7)	3.4 (2.0-5.8)
High	17.9 (17.1-18.7)	2.2 (0.6-8.3)	2.6 (0.9-7.4)	7.0 (4.4-10.8)	7.6 (7.1-8.1)	3.7 (1.0-12.6)	2.7 (0.9-8.0)	2.2 (0.6-8.1)
Used quitline**								
Low	1.5 (1.3-1.7)	0.8 (0.1-4.9)	Not applicable	0	4.0 (3.6-4.4)	4.9 (0.6-29.3)	0	0
Moderate	2.6 (2.3-2.9)	2.3 (0.3-14.8)	Not applicable	0	0.7 (0.5-0.9)	1.8 (0.4-8.5)	1.7 (0.3-10.5)	0
High	5.4 (5.0-5.8)	0	Not applicable	0	1.5 (1.3-1.7)	0	0	0
Used internet**								
Low	6.5 (4.3-9.6)	3.9 (0.6-22.4)	0	4.7 (0.7-26.6)	3.4 (3.0-3.8)	10.4 (2.3-36.1)	1.5 (0.2-10.0)	0
Moderate	10.8 (8.2-14.0)	7.1 (2.3-19.7)	0	0	5.8 (5.3-6.3)	3.3 (1.2-8.8)	4.5 (1.3-14.5)	0
High	17.0 (13.3-21.4)	0	1.2 (0.2-8.7)	0	7.4 (6.9-7.9)	9.8 (2.4-32.8)	0	0
Used smoking cessation service**								
Low	8.8 (6.4-12.0)	2.0 (0.5-7.9)	0	0	2.7 (2.4-3.0)	0	0.8 (0.1-5.4)	0
Moderate	7.7 (5.7-10.5)	0	2.7 (0.7-10.0)	2.0 (0.3-13.7)	3.7 (3.3-4.1)	3.0 (1.2-7.5)	0	4.1 (1.0-16.1)
High	7.7 (5.5-10.7)	0	0	0	5.6 (5.1-6.1)	0	0	0
**Used e-cigarettes to quit**								
Low	57.6 (51.7-63.4)	16.9 (9.8-27.7)	25.0 (13.8-41.2)	14.8 (7.6-26.8)	64.1 (42.9-80.9)	5.5 (0.7-32.2)	13.9 (4.4-36.4)	8.0 (3.3-18.4)
Moderate	50.2 (45.1-55.3)	14.0 (8.0-23.3)	28.1 (16.5-43.6)	21.0 (7.0-48.1)	36.9 (25.4-50.1)	12.3 (6.4-22.1)	11.5 (7.4-17.3)	3.5 (0.7-16.5)
High	50.7 (44.6-56.8)	13.3 (1.8-56.0)	33.4 (19.1-51.7)	11.8 (2.8-38.7)	42.6 (28.2-58.3)	21.4 (12.1-34.9)	2.3 (0.3-14.7)	1.9 (1.2-3.1)
Received advice about quitting from health professional during visit***								
Low	36.0 (32.1-40.1)	40.0 (32.5-48.1)	73.4 (60.5-83.2)	23.6 (16.8-32.1)	24.8 (23.9-25.7)	33.2 (24.4-43.3)	52.3 (41.3-63.2)	48.6 (37.2-60.1)
Moderate	39.4 (35.8-43.0)	39.7 (30.2-50.1)	39.5 (29.5-50.5)	13.9 (7.8-23.5)	22.6 (21.8-23.4)	19.0 (13.8-25.7)	59.0 (51.3-66.2)	42.7 (35.3-50.4)
High	34.5 (30.3-38.9)	33.1 (24.7-42.7)	43.7 (31.9-56.3)	35.5 (20.6-53.9)	19.4 (18.6-20.2)	15.1 (7.4-28.6)	49.9 (36.1-63.6)	46.2 (30.3-62.9)
**Talked about e-cigarettes with health professional**								
Low	5.3 (3.8-7.4)	0.3 (0.3-0.4)	3.0 (1.3-6.7)	10.7 (5.9-18.7)	10.2 (3.9-23.9)	5.4 (4.2-7.0)	8.2 (3.2-19.5)	4.3 (1.8-10.1)
Moderate	6.2 (4.8-7.9)	0.4 (0.1-2.8)	7.2 (3.1-15.7)	8.7 (3.1-22.0)	2.5 (1.2-5.3)	2.8 (0.9-8.1)	9.3 (5.7-14.6)	1.3 (0.4-4.1)
High	10.4 (7.9-13.4)	0	2.8 (1.1-6.8)	5.8 (1.8-17.2)	9.0 (4.7-16.6)	0	3.8 (0.5-22.5)	1.3 (0.2-8.8)

* Netherlands: in the last 12 months. Other countries: as part of your last quit attempt.

** Netherlands: in the last 6 months. Other countries: as part of your last quit attempt.

*** Netherlands: in the last 6 months. Other countries: in the last 12 months.

**Table 5:** Stratified analyses by age on reported quitting activity and use of cessation assistance in 2016 across eight European countries (weighted results)

	*England (n=3536) % (95%CI)*	*Germany (n=1003) % (95%CI)*	*Greece (n=1000) % (95%CI)*	*Hungary (n=1000) % (95%CI)*	*Netherlands (n=1136) % (95%CI)*	*Poland (n=1006) % (95%CI)*	*Romania (n=1001) % (95%CI)*	*Spain (n=1001) % (95%CI)*
**Made quit attempt in past 12 months**								
18-24 years	50.2 (49.2-51.2)	22.4 (14.9-32.3)	25.2 (14.8-39.6)	8.4 (3.8-17.8)	29.2 (22.9-36.4)	30.2 (21.0-41.3)	29.2 (21.0-39.0)	17.2 (11.1-25.6)
25-39 years	54.6 (53.6-55.6)	21.8 (16.2-28.7)	16.6 (11.4-23.6)	12.1 (8.4-17.2)	40.7 (33.5-48.2)	20.6 (15.3-27.1)	26.2 (20.7-32.7)	17.7 (13.1-23.6)
40-54 years	41.9 (40.9-42.9)	15.3 (11.4-20.3)	12.9 (9.9-16.5)	9.6 (6.5-13.9)	25.5 (19.9-32.0)	8.1 (5.5-11.9)	25.5 (20.4-31.4)	19.2 (12.9-27.7)
55+ years	37.6 (36.7-38.5)	13.5 (9.7-18.7)	13.2 (9.1-18.7)	9.8 (6.7-14.2)	30.1 (25.1-35.7)	15.8 (11.7-20.9)	30.1 (23.6-37.5)	15.0 (10.7-20.7)
**Intends to quit**								
**Within 1 month**								
18-24 years	14.8 (11.4-18.9)	2.0 (0.9-4.3)	3.1 (0.9-10.0)	2.5 (0.6-9.7)	4.2 (2.1-8.5)	5.0 (2.0-12.3)	8.2 (3.7-17.2)	4.1 (1.9-8.9)
25-39 years	18.5 (15.2-22.4)	7.4 (4.3-12.5)	1.7 (0.6-4.7)	1.9 (0.8-4.6)	11.6 (7.2-18.0)	3.0 (1.4-6.5)	4.1 (2.3-7.3)	2.7 (1.3-5.4)
40-54 years	11.9 (9.5-14.7)	3.0 (1.6-5.8)	2.3 (1.1-4.7)	2.3 (1.1-4.8)	5.9 (3.1-10.9)	4.6 (2.2-9.3)	10.0 (6.2-15.6)	6.2 (3.6-10.6)
55+ years	10.7 (8.4-13.5)	2.6 (1.3-5.1)	1.5 (0.8-3.0)	2.3 (0.7-7.4)	6.7 (3.7-11.8)	5.2 (2.6-10.0)	6.7 (3.6-12.2)	5.6 (3.1-10.1)
**Within 6 months**								
18-24 years	21.5 (17.6-26.1)	3.4 (1.0-11.0)	5.2 (2.3-11.3)	7.5 (3.0-17.3)	18.8 (13.2-26.0)	14.8 (8.7-24.0)	8.9 (5.0-15.3)	4.7 (2.3-9.6)
25-39 years	32.4 (28.2-36.9)	10.9 (7.9-15.0)	5.1 (3.0-8.6)	10.5 (7.0-15.4)	26.6 (19.6-35.0)	11.5 (7.7-16.7)	9.3 (5.9-14.1)	8.9 (6.1-13.0)
40-54 years	26.7 (23.4-30.3)	7.0 (4.3-11.4)	8.1 (6.0-11.0)	7.7 (5.1-11.5)	21.5 (15.9-28.4)	9.5 (6.1-14.4)	8.2 (5.4-12.2)	7.9 (5.2-11.8)
55+ years	19.1 (16.2-22.5)	6.2 (3.2-11.6)	5.2 (2.6-10.2)	5.9 (3.6-9.4)	21.9 (16.9-27.9)	6.4 (4.0-10.2)	12.0 (7.7-18.2)	9.7 (6.2-14.7)
**Beyond 6 months**								
18-24 years	40.4 (35.4-45.7)	57.0 (42.7-70.1)	34.8 (23.8-47.7)	30.3 (18.9-44.8)	64.1 (56.1-71.4)	27.3 (17.0-40.8)	27.2 (19.2-37.1)	24.7 (15.4-37.1)
25-39 years	32.6 (28.4-37.0)	46.6 (39.3-54.0)	37.6 (28.8-47.4)	19.6 (14.8-25.6)	48.4 (40.3-56.6)	27.0 (20.9-34.1)	38.5 (30.9-46.6)	22.5 (16.6-29.8)
40-54 years	34.4 (30.7-38.4)	48.2 (41.6-54.9)	30.5 (24.5-37.3)	20.2 (15.5-25.9)	51.0 (43.3-58.8)	27.6 (21.2-35.0)	38.4 (31.3-46.1)	24.8 (19.3-31.4)
55+ years	31.1 (27.5-35.0)	40.3 (33.2-47.9)	28.1 (22.7-34.3)	22.8 (17.2-29.6)	47.3 (40.9-53.8)	27.4 (21.6-34.1)	41.7 (34.1-49.6)	21.7 (13.7-32.5)
**Not at all**								
18-24 years	23.3 (19.0-28.1)	37.7 (25.8-51.2)	56.9 (44.3-68.6)	59.8 (46.3-71.9)	12.9 (8.0-19.9)	52.8 (42.4-63.0)	55.7 (44.6-66.3)	66.4 (56.1-75.4)
25-39 years	16.5 (13.4-20.1)	35.1 (27.0-44.1)	55.5 (45.5-65.1)	68.0 (60.7-74.5)	13.4 (8.3-21.0)	58.5 (51.6-65.2)	48.2 (39.9-56.5)	65.8 (57.6-73.2)
40-54 years	27.0 (23.5-30.7)	41.7 (33.8-50.1)	59.1 (51.9-65.9)	69.8 (63.2-75.7)	21.6 (15.9-28.8)	58.4 (50.5-65.8)	43.4 (36.2-50.9)	61.0 (54.1-67.5)
55+ years	39.1 (35.2-43.2)	50.9 (42.1-59.6)	65.1 (58.0-71.6)	69.0 (61.4-75.7)	24.1 (19.2-29.9)	61.0 (53.1-68.4)	39.6 (31.4-48.4)	63.1 (52.7-72.4)
Used medication*								
18-24 years	15.6 (14.9-16.3)	5.2 (2.1-12.3)	4.0 (0.9-15.8)	0	1.9 (1.6-2.2)	6.3 (3.1-12.4)	4.1 (1.0-15.1)	0
25-39 years	16.4 (15.7-17.1)	2.0 (0.8-5.1)	0.8 (0.2-3.7)	2.4 (1.1-5.3)	11.1 (10.5-11.7)	6.9 (4.2-11.3)	2.5 (1.2-5.1)	2.6 (0.9-7.1)
40-54 years	17.9 (17.1-18.7)	1.4 (0.7-2.9)	1.5 (0.5-4.2)	1.9 (0.8-4.8)	10.0 (9.4-10.6)	2.0 (1.0-3.8)	4.3 (2.2-8.3)	3.2 (1.6-6.4)
55+ years	14.1 (13.4-14.8)	1.4 (0.7-2.8)	1.3 (0.5-3.6)	1.0 (0.3-4.0)	4.9 (4.5-5.3)	4.4 (2.3-8.5)	1.3 (0.5-3.0)	1.7 (0.8-3.3)
Used quitline**								
18-24 years	4.4 (4.0-4.8)	0	Not applicable	0	1.0 (0.8-1.2)	3.7 (0.5-22.4)	0	0
25-39 years	4.2 (3.8-4.6)	0	Not applicable	0	0.5 (0.4-0.6)	1.5 (0.4-6.3)	0	0
40-54 years	1.7 (1.4-2.0)	0	Not applicable	0	2.5 (2.2-2.8)	4.0 (0.6-23.8)	3.7 (0.5-22.0)	0
55+ years	1.0 (0.8-1.2)	5.6 (1.3-21.6)	Not applicable	0	2.1 (1.8-2.4)	0	0	0
Used internet**								
18-24 years	20.7 (15.7-26.9)	0	0	0	3.0 (2.7-3.3)	12.1 (2.8-39.9)	12.1 (2.5-42.9)	0
25-39 years	13.5 (9.9-18.2)	7.1 (2.9-16.3)	0	0	9.2 (8.6-9.8)	8.3 (3.3-19.3)	0.6 (0.1-4.3)	0
40-54 years	7.3 (5.0-10.6)	5.5 (1.4-19.2)	1.2 (0.2-8.3)	0	6.6 (6.1-7.1)	2.8 (0.4-17.6)	3.7 (0.5-22.0)	0
55+ years	1.6 (0.7-3.5)	3.0 (0.8-11.5)	0	12.3 (1.8-51.7)	3.3 (2.9-3.7)	0	0	0
Used smoking cessation service**								
18-24 years	8.8 (5.5-13.8)	9.0 (3.7-19.9)	4.4 (0.7-23.1)	0	2.5 (2.2-2.8)	0	0	0
25-39 years	7.8 (5.2-11.5)	0	0	0	4.8 (4.4-5.2)	1.5 (0.4-6.2)	0	2.2 (0.5-8.8)
40-54 years	9.2 (6.5-13.0)	0	0	1.9 (0.3-12.9)	4.3 (3.9-4.7)	1.7 (0.2-11.8)	0	3.7 (0.5-21.9)
55+ years	8.2 (5.2-12.5)	0	2.4 (0.3-15.5)	0	3.9 (3.5-4.3)	4.2 (1.0-16.5)	1.1 (0.2-7.4)	0
**Used e-cigarettes to quit**								
18-24 years	44.7 (37.3-52.4)	28.0 (12.4-51.7)	15.7 (3.3-50.6)	24.8 (2.7-79.7)	44.5 (25.5-65.2)	15.9 (5.2-39.5)	8.3 (2.6-23.4)	0
25-39 years	51.4 (44.7-58.0)	13.5 (5.5-29.3)	32.3 (19.4-48.8)	24.1 (12.2-42.1)	34.9 (19.7-53.9)	19.9 (12.4-30.3)	12.4 (4.8-28.6)	1.1 (0.2-7.6)
40-54 years	58.2 (51.2-64.8)	13.6 (6.9-25.2)	28.4 (15.7-45.9)	13.7 (5.2-31.4)	52.7 (35.1-69.6)	4.8 (1.5-14.7)	12.0 (5.2-25.4)	9.8 (4.2-21.4)
55+ years	51.1 (43.7-58.5)	16.8 (8.3-31.2)	31.9 (18.7-48.8)	2.7 (0.4-16.9)	44.2 (29.6-59.8)	5.5 (1.6-17.2)	9.2 (3.9-20.1)	3.3 (0.8-12.1)
Received advice about quitting from health professional during visit***								
18-24 years	28.9 (23.4-35.1)	19.1 (15.2-23.7)	38.7 (14.4-70.3)	17.5 (10.0-28.9)	9.9 (9.3-10.5)	0	47.5 (32.4-63.0)	32.2 (23.4-42.5)
25-39 years	37.3 (31.8-43.1)	27.3 (18.3-38.6)	34.6 (24.4-46.3)	18.3 (11.0-28.8)	25.2 (24.3-26.1)	15.4 (9.2-24.6)	51.6 (43.1-60.1)	33.4 (23.5-45.1)
40-54 years	38.7 (34.3-43.4)	41.2 (33.8-49.1)	54.5 (41.8-66.7)	18.3 (12.3-26.5)	26.7 (25.8-27.6)	21.7 (16.8-27.6)	55.2 (45.2-64.9)	50.6 (42.8-58.3)
55+ years	44.0 (39.6-48.5)	47.0 (36.8-57.5)	64.7 (46.5-79.4)	28.4 (20.7-37.8)	19.0 (18.2-19.8)	28.3 (21.8-36.0)	69.4 (60.1-77.3)	56.9 (46.1-67.1)
**Talked about e-cigarettes with health professional**								
18-24 years	9.0 (6.2-12.9)	0	0	11.3 (8.3-15.1)	4.8 (1.3-16.5)	0	22.7 (15.0-32.8)	0
25-39 years	7.3 (5.2-10.1)	0	1.8 (0.2-12.0)	9.8 (3.4-25.2)	5.0 (1.4-15.8)	0.4 (0.1-3.0)	3.0 (0.9-9.4)	1.9 (0.6-5.8)
40-54 years	5.7 (4.0-8.1)	0.7 (0.1-5.2)	7.6 (4.3-13.0)	4.9 (2.4-10.0)	6.7 (2.7-15.6)	4.9 (3.1-7.5)	8.9 (3.7-19.9)	1.1 (0.5-2.4)
55+ years	5.6 (4.0-7.9)	0.9 (0.3-2.4)	5.0 (2.0-12.1)	14.9 (7.2-28.2)	7.1 (3.8-13.0)	3.7 (0.8-14.7)	8.9 (4.5-16.9)	8.2 (4.8-13.6)

* Netherlands: in the last 12 months. Other countries: as part of your last quit attempt.

** Netherlands: in the last 6 months. Other countries: as part of your last quit attempt.

*** Netherlands: in the last 6 months. Other countries: in the last 12 months.

Similarly, only a few clear patterns across income groups were identified. In England, more smokers with low income had no intention to quit at all than smokers with high income. In Greece and Hungary, more high-income smokers used e-cigarettes to quit smoking than low-income smokers.

There were several important patterns documented based on educational groups. In Hungary, fewer smokers from the low education group made quit attempts than smokers from the high education group. Furthermore, in England, Germany, Greece, and the Netherlands, low educated smokers were more likely to report having no intention to quit at all than more highly educated smokers. Moreover, in Greece and the Netherlands, low educated smokers were more likely to report having received advice about quitting from a health professional compared to higher educated smokers.

In Poland, more younger smokers made quit attempts than older smokers. Furthermore, in England, Germany, the Netherlands, and Spain, more older smokers reported having received advice about quitting from health professionals than younger smokers.

## DISCUSSION

This study involving smokers from eight European countries showed that, in general, smokers had a low intention to quit tobacco use, indicating a significant loss for European public health. For example, in Hungary, Spain, Greece, and Poland, more than half of smokers were not planning to quit at all. Consequently, only a minority of smokers from these countries had made a quit attempt in the previous year. Only in England did almost half of smokers make a quit attempt in the previous year; in contrast, in the other countries, less than one-third of smokers reported making a quit attempt in the previous year.

Use of available cessation assistance was generally low across countries. Quitlines and local smoking cessation services were almost never used. Indeed, in Greece, Hungary, and Spain, quitlines were not used at all, which is likely to be a reflection of the absence of such services (in Greece there are no quitlines) or very low awareness and/or capacity of quitlines in Hungary and Spain (quitlines are available in a few regions within Spain). Furthermore, only very few smokers who visited a health professional reported having received advice about quitting in several countries sampled, including Poland, Hungary, and the Netherlands. Only in Romania and Greece did over half of smokers who were seen by a health professional report that they received advice to quit smoking.

Our results are similar to the results identified within the secondary analysis of the 2017 Eurobarometer data, in which it was noted that the majority of attempts to quit smoking in the EU are made without any cessation assistance. There was significant heterogeneity between EU countries with regard to the approach to smoking cessation and noted quit attempts; a fact that is closely linked also to implemented and comprehensive smoking cessation policies^19^.

The findings of our study have several implications for policy and practice. First, an important target would be to motivate smokers to make quit attempts. In all countries except England, more than two-thirds of smokers had not made a quit attempt in the past year and were also not planning to do so soon. Educational campaigns regarding the benefits of smoking cessation as well as policy interventions (e.g. taxation) may be important investments in countries where low rates of quitting intentions are reported^37,38^. For example, in Greece where intentions to quit are lowest, smoke-free public place policies are not enforced by the government^39^.

Second, it is important to promote the use of evidence-based cessation support among smokers, in particular those motivated to make a quit attempt. Previous research has shown that smokers may be unaware of the effectiveness of cessation methods or underestimate their benefits^40-42^, which might be reflected in the high prevalence of unassisted quitting^16,43^. It is notable that the most popular cessation aids in our study were e-cigarettes, although the evidence for their effectiveness is modest. It is possible that this popularity is due to their consumer appeal and, prior to implementation of the European Union Tobacco Products Directive, much greater marketing of these products, which is less prominent for other aids in most countries. Increasing use of evidence-based smoking cessation aids is an important target for supporting short- and long-term cessation among smokers, and can be achieved by public health and policy-based interventions.

Third, our findings suggest that health professionals should play a more active role in the smoking cessation process^44^. Several barriers have been identified in previous studies, such as lack of awareness of effective methods, resistance against preventive tasks, communication with the patient, healthcare organizational factors, absence of remuneration for health professionals, and difficulties to apply appropriate frameworks^45-48^. As a next step, more attention should be paid to research on how effective strategies can be implemented in order to find the best ways to improve the existing smoking cessation infrastructure in each country, in particular to reach younger smokers.

Fourth, quitting activity and use of cessation assistance was in general higher in England than in the other countries in our study. The National Health Service in England has made a large investment in cessation through the Stop Smoking Services, the National Centre for Smoking Cessation and Training, cost coverage for smoking cessation medication, and progressive tobacco policy initiatives. For example, West et al.^49^ have shown that reimbursement for smoking cessation medication had a major impact on rates of medication use. It is important to examine whether and how specific measures that are implemented in England could be transferred to other countries, for example, promotion and education about the effectiveness of cessation assistance and the implementation of a national network of smoking cessation services, although recently there has been disinvestment in the services in England. Notably, a recent study with ITC 4CV1 data conducted in Australia, Canada, England, and the US, revealed that smokers from Australia, Canada and the US had even higher rates of advice to quit smoking than smokers from England^50^. This strengthens the conclusion that there is much more potential for many European countries to improve their smoking cessation infrastructure.

Fifth, we found that the subgroup of lower educated smokers needs specific attention as they made fewer quit attempts and had lower intention to quit. This is in line with previous research that identified low education smokers as particularly vulnerable and highly dependent^20,21,51,52^. Educational campaigns to inform this sub-population of smokers about different methods for smoking cessation should be developed. In addition, measures to increase intention to quit among this sub-population of smokers should be implemented, and health professionals might be informed and educated that this group needs more intensive counseling and follow-up. Furthermore, health professionals should be trained to acquire specific skills to be able to support these smokers adequately.

Finally, several countries need to improve their cessation assistance systems to treat tobacco dependence in agreement with WHO FCTC Article 14^17^. For example, only England and the Netherlands conducted campaigns about smoking cessation in 2016. Furthermore, smoking cessation medication is reimbursed in only half of the countries in this study. Previous research has shown that low cost or free medication can trigger thoughts about quitting and increase the likelihood to make a quit attempt among all socioeconomic subgroups^53-56^. Therefore, other countries should consider reimbursement of medication as well. It is also desirable that countries invest in the development of new strategies that are appropriate in times of mobile applications and social media, such as text messaging, digital interventions, or quit smoking apps. Use of such services could be much higher than, for example, telephone quitlines, particularly among younger smokers.

We acknowledge that this study mainly focused on measures that are included in FCTC Article 14 while other factors can also influence quit attempts and intention to quit, for example, the level of nicotine dependence, smokers’ attitudes towards quitting, and social norms about smoking in a country^23,57,58^. Furthermore, tobacco control policies may affect smoking cessation, including taxation, display bans of tobacco products, smoking restrictions in public places, and mass media campaigns^59,60^. Future research could therefore examine which factors in addition to the cessation aids infrastructure in a country are most important for the cessation process.

### Limitations and strengths

First, it is important to note that the data of our study are cross-sectional, which means that no conclusions about effectiveness of use of cessation assistance can be drawn. Future research should longitudinally examine which assistance methods are associated with successful quitting in the separate countries, an approach that will be employed in the analysis of future waves of the ITC 6E Survey. Second, we could only include current smokers in our study, which means that our results probably underestimate the use of cessation methods and the number of quit attempts. Those who had used cessation methods were more likely to have successfully quit^9,61^, and were thus not included. Third, there were differences between data collection (face-to-face vs web), and some survey questions differed between the EURESTPLUS cohorts and the other two ITC cohorts in the Netherlands and England, which might have affected the comparability of country-specific results. Fourth, the study was limited to eight countries, and findings may not be generalizable to other European countries. Fifth, we did not ask about all evidence-based methods for cessation, for example, text-messaging interventions. Sixth, if we had applied more formal testing for significance, we would have corrected our analyses for multiple testing, which might have led to slightly different conclusions. Finally, we are reliant on self-report, and it is possible that there is a degree of forgetting and falsely attributing past efforts to the period asked about. However, we think it is likely that such recall bias would be similar across countries and that the comparison of differences between countries should not be affected. An important strength of the current study is that relatively large and representative samples of smokers from eight European countries could be analyzed and compared. This enabled us to measure and to interpret the findings of separate countries from a broader perspective than past studies.

## CONCLUSIONS

With the exception of England, quitting activity was low among the eight European countries sampled, particularly among smokers with lower education. The highest rates of assistance use were found in England, where large investments in tobacco control and smoking cessation have been made over an extended period, although there is still significant room for improvement. Use of cessation aids among active smokers was very low in all other countries sampled. Furthermore, advice from health professionals about quitting and about e-cigarettes was low in most countries. The findings of this study document the need for efforts to increase support for cessation assistance and to increase awareness of the need for cessation among smokers in the EU, including efforts to motivate smokers to quit, particularly among smokers with a low educational background. These structural and educational supports for increasing cessation called for by the Guidelines for implementation of Article 14 of the FCTC are urgently needed, not only in the European countries included in this study, but throughout the EU and throughout the world.

## References

[1] World Health Organization (2003). Framework Convention on Tobacco Control.

[2] Raw M, Ayo-Yusuf O, Chaloupka F (2017). Recommendations for the implementation of WHO Framework Convention on Tobacco Control Article 14 on tobacco cessation support. Addiction.

[3] Pine-Abata H, McNeill A, Murray R, Bitton A, Rigotti N, Raw M (2013). A survey of tobacco dependence treatment services in 121 countries. Addiction.

[4] Raw M, Regan S, Rigotti NA, McNeill A (2009). A survey of tobacco dependence treatment services in 36 countries. Addiction.

[5] Nilan K, Raw M, McKeever TM, Murray RL, McNeill A (2017). Progress in implementation of WHO FCTC Article 14 and its guidelines: a survey of tobacco dependence treatment provision in 142 countries. Addiction.

[6] Raw M, Mackay J, Reddy S (2016). Time to take tobacco dependence treatment seriously. Lancet.

[7] West R, Raw M, McNeill A (2015). Health-care interventions to promote and assist tobacco cessation: a review of efficacy, effectiveness and affordability for use in national guideline development. Addiction.

[8] Aveyard P, Begh R, Parsons A, West R (2012). Brief opportunistic smoking cessation interventions: a systematic review and meta-analysis to compare advice to quit and offer of assistance. Addiction.

[9] Patnode CD, Henderson JT, Thompson JH, Senger CA, Fortmann SP, Whitlock EP (2015). U.S. Preventive Services Task Force Evidence Syntheses, Formerly Systematic Evidence Reviews. Behavioral Counseling and Pharmacotherapy Interventions for Tobacco Cessation in Adults, Including Pregnant Women: A Review of Reviews for the U.S. Preventive Services Task Force.

[10] Stead LF, Koilpillai P, Fanshawe TR, Lancaster T (2016). Combined pharmacotherapy and behavioural interventions for smoking cessation. The Cochrane Database of Systematic Reviews.

[11] Lam C, West A (2015). Are electronic nicotine delivery systems an effective smoking cessation tool?. Canadian Journal of Respiratory Therapy.

[12] McRobbie H, Bullen C, Hartmann-Boyce J, Hajek P (2014). Electronic cigarettes for smoking cessation and reduction. The Cochrane Database of Systematic Reviews.

[13] Ghorai K, Akter S, Khatun F, Ray P (2014). mHealth for Smoking Cessation Programs: A Systematic Review. Journal of Personalized Medicine.

[14] Warner KE, Mendez D (2018). E-cigarettes: Comparing the Possible Risks of Increasing Smoking Initiation with the Potential Benefits of Increasing Smoking Cessation. Nicotine & Tobacco Research.

[15] Stead LF, Carroll AJ, Lancaster T (2017). Group behaviour therapy programmes for smoking cessation. The Cochrane Database of Systematic Reviews.

[16] Filippidis FT, Gerovasili V, Vardavas CI, Agaku IT, Tountas Y (2014). Determinants of use of smoking cessation aids in 27 European countries. Preventive Medicine.

[17] World Health Organization Guidelines for implementation of Article 14.

[18] Borland R, Li L, Driezen P (2012). Cessation assistance reported by smokers in 15 countries participating in the International Tobacco Control (ITC) policy evaluation surveys. Addiction.

[19] Filippidis FT, Laverty AA, Mons U, Jimenez-Ruiz C, Vardavas CI (2018). Changes in smoking cessation assistance in the European Union between 2012 and 2017: pharmacotherapy versus counselling versus e-cigarettes. Tobacco Control.

[20] Hiscock R, Bauld L, Amos A, Fidler JA, Munafo M (2012). Socioeconomic status and smoking: a review. Annals of the New York Academy of Sciences.

[21] Reid JL, Hammond D, Boudreau C, Fong GT, Siahpush M (2010). Socioeconomic disparities in quit intentions, quit attempts, and smoking abstinence among smokers in four western countries: findings from the International Tobacco Control Four Country Survey. Nicotine & Tobacco Research.

[22] Kasza KA, Hyland AJ, Borland R (2013). Effectiveness of stop-smoking medications: findings from the International Tobacco Control (ITC) Four Country Survey. Addiction.

[23] Hyland A, Li Q, Bauer JE, Giovino GA, Steger C, Cummings KM (2004). Predictors of cessation in a cohort of current and former smokers followed over 13 years. Nicotine & Tobacco Research.

[24] ITC Project International Tobacco Control Policy Evaluation Project.

[25] Thompson ME, Fong GT, Hammond D (2006). Methods of the International Tobacco Control (ITC) Four Country Survey. Tobacco Control.

[26] Fong GT, Cummings KM, Borland R (2006). The conceptual framework of the International Tobacco Control (ITC) Policy Evaluation Project. Tobacco Control.

[27] Vardavas CI, Bécuwe N, Demjén T (2018). Study Protocol of European Regulatory Science on Tobacco (EURESTPLUS): Policy implementation to reduce lung disease. Tobacco Induced Diseases.

[28] European Commission Special Eurobarometer 458. “Attitudes of Europeans towards tobacco and electronic cigarettes”.

[29] Joossens L, Raw M The Tobacco Control Scale 2016 in Europe.

[30] Fong GT, Thompson ME, Boudreau C (2018). The Conceptual Model and Methods of Wave 1 (2016) of the EUREST-PLUS ITC 6 European Countries Survey. Tobacco Induced Diseases.

[31] ITC Project International Tobacco Control Policy Evaluation Survey (ITC). Four Country Project. Waves 2-8 Technical Report.

[32] ITC Project Technical Report United Kingdom.

[33] Nagelhout GE, Willemsen MC, Thompson ME, Fong GT, Van den Putte B, De Vries H (2010). Is web interviewing a good alternative to telephone interviewing? Findings from the International Tobacco Control (ITC) Netherlands survey. BMC Public Health.

[34] Zethof D, Nagelhout GE, De Rooij M (2016). Attrition analysed in five waves of a longitudinal yearly survey of smokers: findings from the ITC Netherlands survey. European Journal of Public Health.

[35] ITC Project Technical Report Netherlands.

[36] Hummel K, Candel M, Nagelhout GE (2017). Construct and predictive validity of three measures of intention to quit smoking: Findings from the International Tobacco Control (ITC) Netherlands Survey. Nicotine & Tobacco Research.

[37] Durkin S, Brennan E, Wakefield M (2012). Mass media campaigns to promote smoking cessation among adults: an integrative review. Tobacco Control.

[38] Ross H, Blecher E, Yan L, Hyland A (2011). Do cigarette prices motivate smokers to quit? New evidence from the ITC survey. Addiction.

[39] Filippidis FT, Tzoulaki I (2016). Greece giving up on tobacco control. Addiction.

[40] Hammond D, McDonald PW, Fong GT, Borland  R (2004). Do smokers know how to quit? Knowledge and perceived effectiveness of cessation assistance as predictors of cessation behaviour. Addiction.

[41] Fu SS, Burgess D, van Ryn M, Hatsukami DK, Solomon J, Joseph AM (2007). Views on smoking cessation methods in ethnic minority communities: a qualitative investigation. Preventive Medicine.

[42] Papadakis S, Hurtubise R, Galley S Tobacco users knowledge, attitudes, and barriers regarding quit smoking support: a cross-sectional survey.

[43] Edwards SA, Bondy SJ, Callaghan RC, Mann RE (2014). Prevalence of unassisted quit attempts in population-based studies: a systematic review of the literature. Addictive Behaviors.

[44] Papadakis S, McDonald P, Mullen KA, Reid R, Skulsky K, Pipe A (2010). Strategies to increase the delivery of smoking cessation treatments in primary care settings: a systematic review and meta-analysis. Preventive Medicine.

[45] van Rossem C, Spigt MG, Kleijsen JR, Hendricx M, van Schayck CP, Kotz D (2015). Smoking cessation in primary care: Exploration of barriers and solutions in current daily practice from the perspective of smokers and healthcare professionals. The European Journal of General Practice.

[46] Johnston KN, Young M, Grimmer-Somers KA, Antic R, Frith PA (2011). Why are some evidence-based care recommendations in chronic obstructive pulmonary disease better implemented than others? Perspectives of medical practitioners. International Journal of Chronic Obstructive Pulmonary Disease.

[47] van Eerd EAM, Bech Risor M, Spigt M (2017). Why do physicians lack engagement with smoking cessation treatment in their COPD patients? A multinational qualitative study. NPJ Primary Care Respiratory Medicine.

[48] Martinez C (2009). Barriers and challenges of implementing tobacco control policies in hospitals: applying the institutional analysis and development framework to the Catalan Network of Smoke-Free Hospitals. Policy, Politics & Nursing Practice.

[49] West R, DiMarino ME, Gitchell J, McNeill A (2005). Impact of UK policy initiatives on use of medicines to aid smoking cessation. Tobacco Control.

[50] Gravely S, Thrasher JF, Cummings KM Discussions between health professionals and smokers about e-cigarettes: Results from the ITC Four Country Tobacco and E-Cigarette Survey.

[51] Giskes K, Kunst AE, Benach J (2005). Trends in smoking behaviour between 1985 and 2000 in nine European countries by education. Journal of Epidemiology and Community Health.

[52] Siahpush M, McNeill A, Borland R, Fong GT (2006). Socioeconomic variations in nicotine dependence, self-efficacy, and intention to quit across four countries: findings from the International Tobacco Control (ITC) Four Country Survey. Tobacco Control.

[53] Hummel K, Nagelhout GE, Willemsen MC (2015). Trends and socioeconomic differences in policy triggers for thinking about quitting smoking: Findings from the International Tobacco Control (ITC) Europe Surveys. Drug and Alcohol Dependence.

[54] Kasza KA, Hyland AJ, Borland R (2017). Cross-country comparison of smokers’ reasons for thinking about quitting over time: findings from the International Tobacco Control Four Country Survey (ITC-4C), 2002-2015. Tobacco Control.

[55] van den Brand FA, Nagelhout GE, Reda AA (2017). Healthcare financing systems for increasing the use of tobacco dependence treatment. The Cochrane Database of Systematic Reviews.

[56] Trofor AC, Man MA, Marginean C, Dumitru F, Trofor L (2016). Smoking cessation for free: outcomes of a study of three Romanian clinics. Open Medicine.

[57] Hyland A, Borland R, Li Q (2006). Individual-level predictors of cessation behaviours among participants in the International Tobacco Control (ITC) Four Country Survey. Tobacco Control.

[58] Zhang X, Cowling DW, Tang H (2010). The impact of social norm change strategies on smokers’ quitting behaviours. Tobacco Control.

[59] Feliu A, Filippidis FT, Joossens L (2018). Impact of tobacco control policies on smoking prevalence and quit ratios in 27 European Union countries from 2006 to 2014. Tobacco Control.

[60] Levy DT, Tam J, Kuo C, Fong GT, Chaloupka F (2018). The Impact of Implementing Tobacco Control Policies: The 2017 Tobacco Control Policy Scorecard. Journal of Public Health Management and Practice.

[61] Hartmann-Boyce J, Chepkin SC, Ye W, Bullen C, Lancaster T (journal). Nicotine replacement therapy versus control for smoking cessation. The Cochrane Database of Systematic Reviews.

