# Supplementary material for: Quitting activity and use of cessation assistance reported by smokers in eight European countries: Findings from the EUREST-PLUS ITC Europe Surveys
Source: Tob Induc Dis. 2018 Dec 21;16:A6. doi: 10.18332/tid/98912 (PMC6659556; doi:10.18332/tid/98912)
Supplement: Supplementary file 1 [file TID-16-A6-s1.pdf]

Supplementary Table S1. Country ranking for quitting activity and used cessation assistance

	Country ranking
Made quit attempt in past 12 months	1. England<br>2. The Netherlands<br>3. Romania<br>4. Spain<br>5. Germany<br>6. Poland<br>7. Greece<br>8. Hungary
Intends to quit within 1 months	1. England<br>2. The Netherlands<br>3. Romania<br>4. Spain<br>5. Poland<br>6. Germany<br>7. Hungary<br>8. Greece
Used medication*	1. England<br>2. The Netherlands<br>3. Poland<br>4. Romania<br>5. Spain<br>6. Germany<br>7. Hungary<br>8. Greece
Used quitline**	1. England<br>2. Poland<br>3. The Netherlands<br>4. Germany<br>5. Romania<br>6. Greece<br>Hungary<br>Spain
Used internet**	1. England<br>2. The Netherlands<br>Poland<br>4. Germany<br>5. Romania<br>6. Hungary<br>7. Greece<br>8. Spain
Used smoking cessation service**	1. England<br>2. The Netherlands<br>3. Spain<br>4. Poland<br>5. Greece<br>6. Germany<br>7. Hungary<br>8. Romania
Used e-cigarettes to quit	1. England<br>2. The Netherlands<br>3. Greece<br>4. Hungary<br>5. Germany<br>6. Poland<br>7. Romania<br>8. Spain

Received advice about quitting from health professional during visit***	1.	Romania
	2.	Greece
	3.	Spain
	4.	Germany
	5.	England
	6.	The Netherlands
	7.	Hungary
	8.	Poland
Talked about e-cigarettes with health professional	1.	Hungary
	2.	Romania
	3.	England
	4.	The Netherlands
	5.	Greece
	6.	Poland
	7.	Spain
	8.	Germany

*Netherlands: in the last 12 months. Other countries: as part of your last quit attempt.
**Netherlands: in the last 6 months. Other countries: as part of your last quit attempt.
***Netherlands: in the last 6 months. Other countries: in the last 12 months.

© 2018 Hummel K.
